# Influences of Infrared Dye Content and Laser Energy Density on Laser Propulsion Performance of ADN-Based Liquid Propellants

**DOI:** 10.3390/mi16010059

**Published:** 2024-12-31

**Authors:** Lizhi Wu, Jinle Cao, Jingyuan Zhang, Jingwei Zeng, Yue Pan

**Affiliations:** 1 School of Chemistry and Chemical Engineering, Nanjing University of Science and Technology, Nanjing 210094, China; 2 Micro-Nano Energetic Devices Key Laboratory of MIIT, Nanjing 210094, China; 3 Institute of Space Propulsion, Nanjing University of Science and Technology, Nanjing 210094, China; 4 Anhui Hongxing Mechancal & Electrical Tech. Co., Ltd., Hefei 231635, China; 5 The 41 Institute of the Sixth Academy of China Aerospace Science and Industry, Hohhot 010010, China

**Keywords:** laser ablation liquid propulsion, ammonium dinitramide (ADN), liquid propellant, ablation efficiency

## Abstract

Ammonium dinitramide (ADN) is a new green oxidant, which is a kind of high-energy ionic liquid and has been widely used in the field of liquid propulsion. When it is used in laser plasma propulsion, its poor absorption coefficient significantly limits its application. To address the issue, this paper investigates the effects of the content of the infrared dye and the laser energy density on the laser propulsion performance of an ADN-based liquid propellant. The performance of the liquid propellant was tested by the light absorption performance test system and the micro-impulse test system. The results show that the addition of infrared dye can significantly improve the light absorption performance of the liquid matrix. As the content of the infrared (IR) dyes increases from 0.3 wt.% to 0.6 wt.%, the absorption coefficient of the ADN-based liquid propellant increases from 248.84 cm^−1^ to 463.85 cm^−1^, and the absorption depth decreases from 40.20 μm to 21.56 μm. At a laser energy density of 21.60 J·cm^−1^, when the IR dye content increases from 0.3 wt.% to 0.6 wt.%, the specific impulse increases from 26.43 s to 54.43 s and the ablation efficiency increases from 4.32% to 18.21%. Significantly luminous plasma appears in the ablation plume at higher laser energy densities, accompanied by higher-velocity plasma shock waves. Compared to the factor of the infrared dye content, the laser energy density contributes more to the ablation efficiency, especially when the increase in laser energy density promotes the full release of chemical energy from the liquid propellant, which, in turn, also enhances the impulse, impulse coupling coefficient, and the plasma detonation velocity. The results provide an important reference for the design of an energy-containing liquid propellant.

## 1. Introduction

Laser plasma propulsion is a new kind of propulsion technology with the advantages of high specific impulse, low cost, and adjustable thrust. It could be used in the realisations of attitude control, orbit adjustment, and inter-hole debris removal through the plasma plume recoil thrust generated by the interaction of the laser and working medium [1,2,3]. Since the concept of laser propulsion was proposed in 1972 [4], it has attracted extensive attention from scholars at home and abroad. Many researchers have carried out a lot of research on the working medium of laser ablation propulsion, such as various metal materials, polymers, liquids, etc. [5,6,7].

The liquid target is an ideal choice because of their high coupling coefficient and the convenience of a micro supply. At present, liquid propellants that were widely studied include non-energetic working fluid such as water, ethanol [8], and glycerol [9], and energetic working fluid such as poly (azide glycidyl ether) (GAP) [10] and hydroxylamine nitrate [11]. For propellants, energy density refers to the energy carried by a substance per unit volume or unit mass. Obviously, the energetic liquid per unit volume/mass has a higher energy than non-energetic ones, which is beneficial to improving the work capacity per unit mass of propellant. Although the impulse coupling performance of liquid propellant is higher than that of solid and gas, due to the poor absorption performance of the liquid propellant, a large amount of working medium will be wasted through liquid sputtering [12] during laser ablation.

Researchers have carried out a large number of studies to improve the laser absorption properties of propellants by adding nanoparticles and infrared dyes to obtain a better propulsion performance. Li Nanlei et al. [13] studied the laser ablation properties of glycerol doped with different contents of carbon powder, and found that glycerol has the best impulse coupling characteristics when the content of carbon powder is 1%, and the impulse coupling coefficient can reach 1250 mN·s·J^−1^. Zheng Zhiyuan et al. [14,15] carried out a relevant study on water and ink propellant by using the transmission laser ablation mode. It was found that the best impulse characteristics were achieved when the laser focus point was close to the junction of the liquid media and the glass carrier, and the impulse coupling coefficient could reach 1790 N·MW^−1^, with a corresponding specific impulse of 19 s.

Energetic materials have a high energy density and can release a large amount of chemical energy in the process of action. Ammonium dinitramide (ADN), as a high-energy-density green compound with good solubility [16,17,18], has been widely used in the field of solid propulsion and single-component liquid propulsion [19,20,21]. However, the low absorption coefficient is the main problem to solve with regard to energetic liquid media. In this study, an ADN-based liquid propellant was formulated with ADN, 1-Allyl-3-methylimidazolium dicyanamide ([AMIM][DCA]), infrared dye IR1036, and water. Focusing on the laser absorption performance of the propellant, the propulsion performance of this liquid propellant was investigated by using an impulse measurement system built based on a torsion pendulum, and the jet flow field of the laser-ablated liquid propellant was collected by using a patterned shadow system to obtain the laws of influence of the infrared dye and the laser energy density on the laser propulsion performance of the ADN-based liquid propellant.

## 2. Experimental Setup and Methodology

### 2.1. Materials

ADN was selected as the oxidising component of the liquid matrix, and the aqueous solution of ADN was provided by Liming Research Institute of Chemical Industry, in which the ADN content of the solution was 74 wt.%. The ionic liquid [AMIM][DCA] was used as the fuel component of the liquid propellant, which was provided by Shanghai Dibo Biotechnology Co., Shanghai, China. Water-soluble infrared dye IR1036 was selected as the light-absorbing component of the liquid matrix, provided by Changchun Xuancai New Material Co., Changchun, China. When selecting the absorbing material, the first consideration is to have a high absorption coefficient for the 1064 nm-wavelength laser, and the second is to meet the requirement of high solubility in water, because our liquid propellant solvent is water. We tested the absorption coefficients and solubility in water of five infrared dyes, IR1075, IR1036, IR1064, IR1089, and IR-H. Their absorption capacities for 1064 nm pulsed laser were in the order of IR1075 > IR1036 > IR1064 > IR1089 > IR-H (Table 1). Although IR1075 had the best absorption performance, its solubility in water was poor, while IR1036 infrared absorbing dye was second only to IR1075 in terms of laser absorption performance, but it had better solubility in water. The parameters of different infrared dyes are shown in Table 1, which are favourable for the preparation of ADN-based liquid propellant. Finally, IR1036 was selected as the light absorber component of the liquid media.

### 2.2. Preparation Process

The ADN-based liquid propellant in this study consists of ADN, [AMIM][DCA], infrared dye IR1036, and solvent water. In propellant formulation, ADN is the oxidants, and the [AMIM][DCA] anion is also the oxidising agent. Under laser ablation, both components undergo some reduction reaction. The optimal mass percentage of ADN and [AMIM][DCA] in the propellant formulation is 73.4% and 10%, when the stability, flow properties, and propulsion performance of the propellant are best.

**Figure 1:**
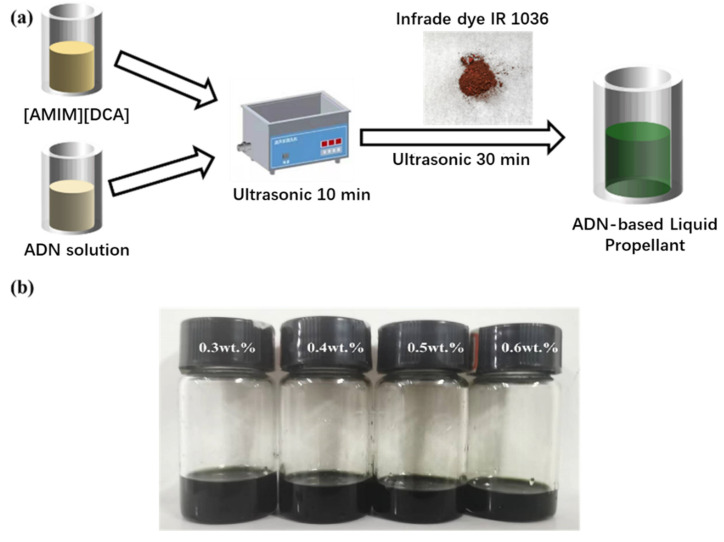
Schematic of ammonium-dinitramide-based propellant preparation (**a**) and ammonium-dinitramide-based propellant doped with different content of infrared dye (**b**).

The preparation process is illustrated in Figure 1a. A specified mass of ADN aqueous solution and ionic liquid [AMIM][DCA] is weighed according to the proportion and subjected to ultrasonic treatment for 10 min. Subsequently, a certain mass of infrared dye IR1036 is weighed and added to the mixed solution of ADN and [AMIM][DCA], which has been homogenised. The mixture is then subjected to ultrasonic treatment for 30 min to fully dissolve the infrared dye in the mixed solution, resulting in a fully dissolved ADN-based liquid propellant, as shown in Figure 1b. The content of [AMIM][DCA] in the ADN-based liquid propellant is 20 wt.%, and the contents of infrared dye are 0.3 wt.%, 0.4 wt.%, 0.5 wt.%, and 0.6 wt.%, respectively.

### 2.3. Optical Setup

#### 2.3.1. Test Method for Absorbance Performance

**Figure 2:**
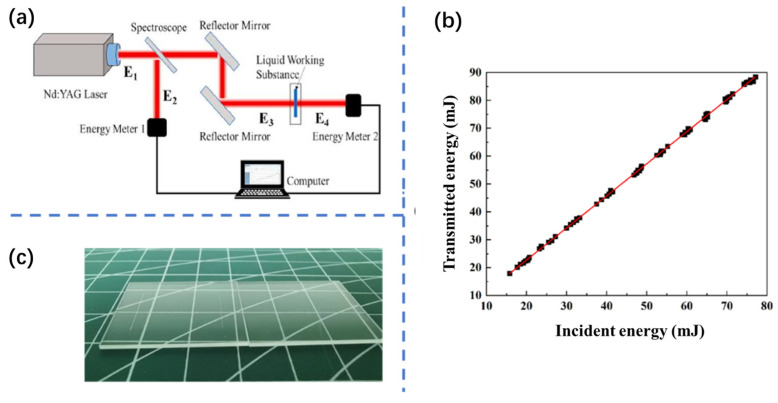
System for testing the absorbance properties of liquid propellant and calibration data ((**a**), schematic diagram; (**b**), calibration data physical diagram; and (**c**), physical diagram of a separable cuvette).

To investigate the laser absorption properties of the liquid propellant, an absorption performance testing system was established as shown in Figure 2a. This system comprises a laser, optical transmission components, a split cuvette (Figure 2c), and energy meters. The laser generates a beam with a wavelength of 1064 nm, a pulse width of 6.5 ns, and a spot diameter of 6 mm. The beam is split into two by a beam splitter; one portion is received by Energy Meter 1 to monitor the laser energy. The energy transmitted through the beam splitter is reflected by a mirror and directed to the split cuvette. Part of the laser energy is absorbed by the liquid propellant within the cuvette, while the remaining energy is transmitted through the liquid and received by Energy Meter 2, provided by Ophir company, North Logan, USA. By calibrating the testing system, the relationship between transmitted energy and reflected energy can be determined, accounting for the reflection and absorption losses caused by the cuvette. The test data in the experiment are all single-pulse test results.

Figure 2b shows the energy calibration of the test system, where *E*_1_ is the energy reflected through the beam splitter and *E*_2_ is the energy transmitted through the beam splitter. After testing and data fitting, the following incident energy calculation formula was obtained:

(1) $$E_{2}={1.15E}_{1}-0.31$$

where $E_{2}$ is the incident laser energy, mJ; and $E_{1}$ is the reflected laser energy, mJ.

Since the incident energy is obtained by Energy Meter 1 and the transmission energy is obtained by Energy Meter 2, the relationship between the two is related to the beam splitter’s splitting ratio, which is approximated to be 53.5:46.5, so the calibrated incident energy is lower than the transmission energy.

#### 2.3.2. Testing Methods for Propulsion Performance

**Figure 3:**
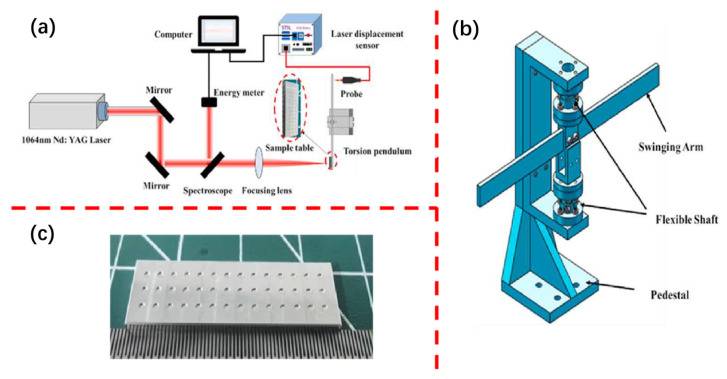
Micro-impulse test system ((**a**), schematic; (**b**), torsion pendulum; and (**c**), aluminum alloy sample tank).

The laser ablation propulsion performance testing apparatus is illustrated in Figure 3. The liquid propellant is contained within an aluminum alloy sample slot with a diameter of 1 mm and a depth of 200 μm (Figure 3c). The laser ablation propulsion performance testing apparatus consists of a laser, an energy meter, a torsion pendulum, a displacement sensor, and optical components. The dynamic model of the torsion pendulum is simplified to a second-order mass-spring-damping model. The relationship between impulse and pendulum arm amplitude is given by Equation (2), with the detailed derivation and calibration process available in reference [22]:

(2) $$I=\frac{J\omega_{0}}{l_{sensor}l_{force}}A_{0}$$

where *J* is the moment of inertia of the torsional pendulum system, kg·m^−2^; $\omega_{0}$ is the natural frequency of the torsion system, s^−1^; $l_{sensor}$ is the distance from the laser displacement sensor to the center of the torsion pivot, m; and $l_{force}$ is the distance between the thrust position of the laser ablation liquid working fluid and the center of the torsion pivot, m. Twist, $l_{sensor}$, and $l_{force}$ parameters can be measured directly. The sensor distance and sample distance parameters of the torsion swing can be directly measured, the measurement accuracy of the laser displacement sensor can reach 0.46 μm, the maximum measurement accuracy of the torsion swing system is 9.48 × 10^−8^ N·s, and the test range is 9.48 × 10^−8^ N·s~3.38 × 10^−4^ N·s.

In the field of laser ablation propulsion, the impulse coupling coefficient, specific impulse, and ablation efficiency are commonly used metrics for evaluating laser propulsion performance. The impulse coupling coefficient is defined as the impulse generated per unit of laser energy ablated from the target material. This coefficient can assess the efficiency of laser energy utilisation in laser ablation thrusters, as expressed in Equation (3):

(3) $$C_{m}=\frac{I}{E}$$

where the impulse generated by the interaction between the laser and the target at *I*, measured by torsional pendulum, N·s; and E is the incident laser energy, J.

The specific impulse represents the impulse generated by the pulsed laser ablation per unit mass of the target and is used to evaluate the utilisation efficiency of the laser ablation thruster, as shown in Equation (4):

(4) $$I_{sp}=\frac{I}{mg}$$

where *m* refers to the mass loss of the target caused by laser ablation, kg. In the laser ablation process, liquid working fluids are accompanied by two main physical processes: vaporisation and liquid sputtering, in which sputtering mass consumption accounts for about 95% of the total mass consumption. g is the acceleration due to gravity, usually 9.80 m/s^2^.

Ablation efficiency refers to the kinetic energy that pulsed laser energy can be converted into the target during the laser ablation process, as shown in Equation (5):

(5) $$\eta =\frac{\frac{1}{2}mV^{2}}{E}=\frac{g}{2}C_{m}I_{sp}=\frac{I^{2}}{2mE}$$

where *V* is the vapor plume velocity generated by laser ablation of the target, m/s. The ablation efficiency is usually calculated directly from the impulse, the incident laser energy, and the ablation mass.

#### 2.3.3. Ablation Flow Field Test

The Schlieren system (Sichuan Jiuyuan Optoelectronics Co., Ltd., Mianyang, China) and a high-speed camera (Phantom V2012, Wayne, NJ, USA) were used to capture transient flow field images of the laser ablation of ADN-based liquid propellant. The flow field was analyzed with a focus on the shock wave evolution process images of ADN solution containing 0.6 wt.% infrared dye under laser ablation at different energy densities.

## 3. Results and Discussion

### 3.1. Laser Absorption Properties

**Figure 4:**
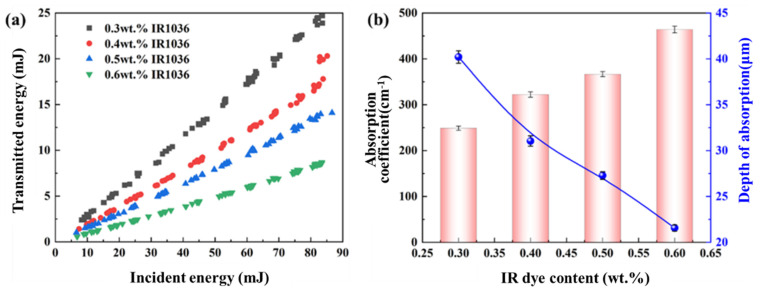
Laser absorption properties of liquid propellant ((**a**), incident energy versus transmitted energy; and (**b**), infrared dye content versus absorption coefficient, depth of absorption).

The infrared dye exhibits good solubility in water and significantly enhances the light absorption properties of the ADN-based liquid propellant when added. The relationship between the incident energy and transmitted energy of the ADN-based liquid propellant is shown in Figure 4a. There is a linear relationship between the incident energy and transmitted energy; as the incident energy increases, the transmitted energy also increases. At the same incident energy, an increase in the infrared dye content in the liquid propellant results in a decrease in transmitted energy, indicating that the absorption performance of the liquid propellant improves with a higher infrared dye content.

The absorption coefficient and absorption depth of the ADN-based liquid propellant with varying infrared dye contents are shown in Figure 4b. When the infrared dye content is 0.3 wt.%, the absorption coefficient of the liquid propellant is 248.84 cm^−1^. As the infrared dye content increases to 0.6 wt.%, the absorption coefficient rises to 463.85 cm^−1^, while the absorption depth decreases from 40.20 μm to 21.56 μm. The results indicate that the addition of infrared dye significantly enhances the absorption capability of the ADN-based liquid propellant for the 1064 nm pulsed laser, with a higher infrared dye content leading to a higher absorption coefficient and a smaller absorption depth.

### 3.2. Propulsion Performance

**Figure 5:**
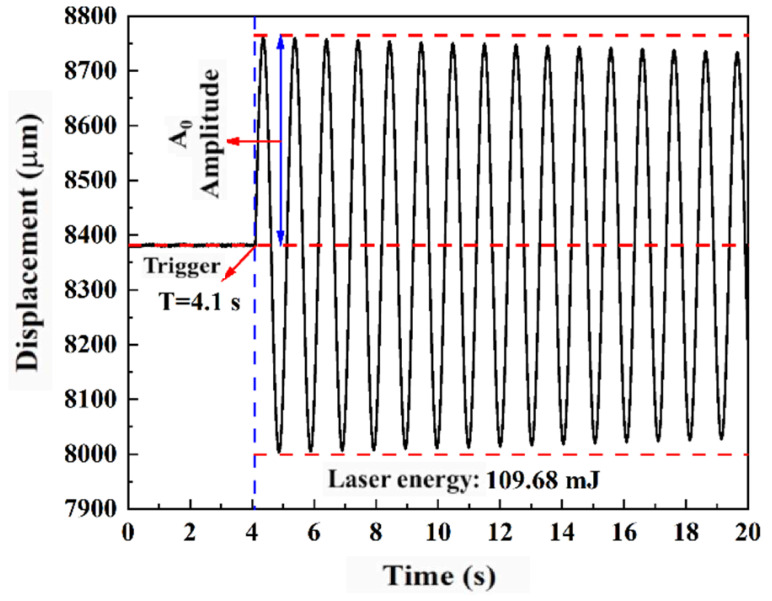
Typical displacement curve of impulse bit test of ADN-based liquid propellant (IR1036 content, 0.6 wt.%).

Figure 5 presents a typical impulse test curve for the ADN-based liquid propellant with an infrared dye content of 0.6 wt.%, using a laser energy of 109.68 mJ. In the initial phase, due to natural environmental vibrations and disturbances, the swing amplitude is 3 μm, oscillating near the equilibrium position. At t = 4.1 s, the pulsed laser irradiates the propellant surface, causing ablation and generating thrust, which results in a maximum swing amplitude of 378.6 μm for the pendulum arm. The amplitude gradually decreases due to environmental damping effects.

#### 3.2.1. Effect of Laser Energy Density on Propulsion Performance

**Figure 6:**
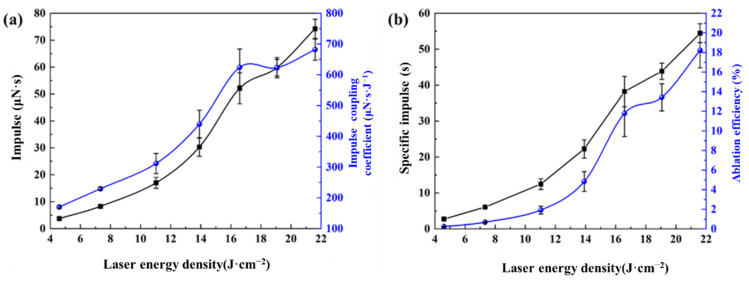
Relationship between the effect of laser energy density on propulsion performance ((**a**) impulse-impulse coupling coefficient; and (**b**) specific impulse-ablation efficiency).

Figure 6 illustrates the effect of the laser energy density on the propulsion performance of the ADN-based liquid propellant, with an infrared dye content of 0.6 wt.% and a laser energy density range of 4.60 J·cm^−2^ to 21.60 J·cm^−2^. At a laser energy density of 4.60 J·cm^−2^, the impulse of the ADN-based liquid propellant is 3.73 μN·s, which increases to 74.22 μN·s as the laser energy density rises to 21.60 J·cm^−2^. The impact of the laser energy density on the impulse coupling coefficient is shown in Figure 6a. As the laser energy density increases, the impulse coupling coefficient also increases, from 170.14 μN·s·J^−1^ at 4.60 J·cm^−2^ to 681.63 μN·s·J^−1^ at 21.60 J·cm^−2^.

The relationship between the specific impulse and ablation efficiency with varying laser energy densities is depicted in Figure 6b. The specific impulse is lowest at 2.73 s when the laser energy density is 4.60 J·cm^−2^. As the laser energy density increases, the specific impulse rises to 6.05 s, 12.46 s, 22.24 s, 38.21 s, and 43.86 s, reaching 54.43 s at 21.60 J·cm^−2^. The ablation efficiency is lowest at 0.23% for 4.60 J·cm^−2^, and it increases with higher energy densities to 0.68%, 1.92%, 4.83%, 11.78%, and 13.42%, reaching 18.21% at 21.60 J·cm^−2^.

The study results indicate that, as the laser energy density increases, the impulse, impulse coupling coefficient, specific impulse, and ablation efficiency of the ADN-based liquid propellant also increase. This is because, under the same infrared dye content, a higher laser energy density results in greater laser energy absorption per unit volume of liquid propellant, leading to a higher degree of ablation. Consequently, propulsion performance parameters such as impulse and specific impulse increase with a higher laser energy density. The specific impulse and ablation efficiency shown in Figure 6b are relatively low, with a maximum specific impulse of 54.43 s and a maximum ablation efficiency of 18.21%. This is because the ablation mass used in the calculations is based on the charge mass of the liquid propellant, which is 139.13 μg for a charge mass equal to the spot diameter. This charge mass is larger than the actual ablation mass, resulting in a lower calculated specific impulse and ablation efficiency.

#### 3.2.2. Effect of Content of IR Dye on Propulsion Performance

**Figure 7:**
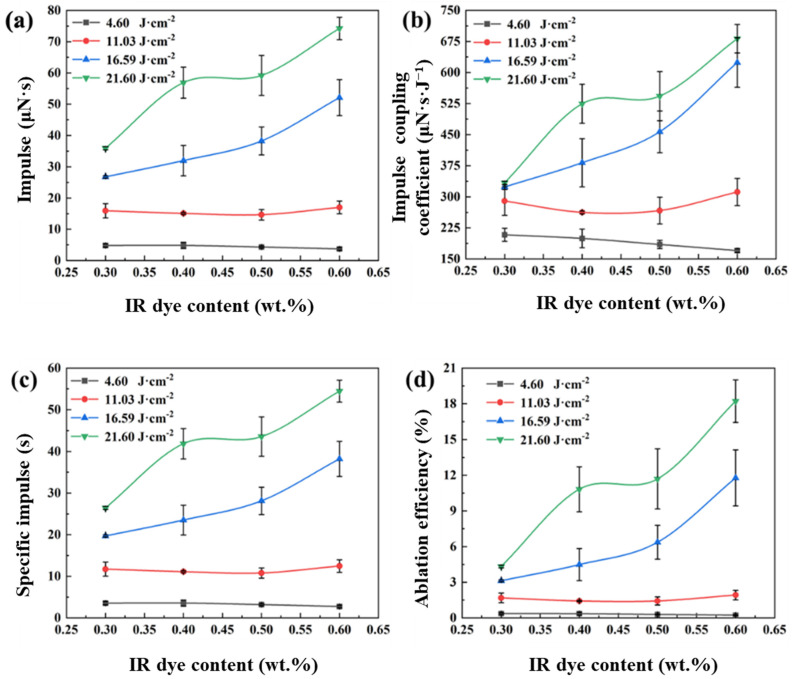
Influence of infrared dye content on propulsion performance ((**a**), impulse; (**b**), impulse coupling coefficient, (**c**), specific impulse; and (**d**), ablation efficiency).

Infrared dye significantly affects the light absorption properties of the ADN-based liquid propellant. A higher infrared dye content enhances the propellant’s light absorption capability, which, in turn, influences its propulsion performance. Figure 7 illustrates the impact of the infrared dye content on the propulsion performance of the liquid propellant, with dye contents of 0.3 wt.%, 0.4 wt.%, 0.5 wt.%, and 0.6 wt.%, and laser energy densities of 4.60 J·cm^−2^, 11.03 J·cm^−2^, 16.59 J·cm^−2^, and 21.60 J·cm^−2^, respectively. As shown in Figure 7a, at lower laser energy densities (4.60 J·cm^−2^ and 11.03 J·cm^−2^), the infrared dye content has a minimal effect on the impulse of the liquid propellant. However, at higher laser energy densities, the impulse of the liquid propellant increases with a higher infrared dye content. Under a laser energy density of 21.60 J·cm^−2^, the impulse of the liquid propellant increases from 35.91 μN·s at 0.3 wt.% to 74.22 μN·s at 0.6 wt.%.

The effect of the infrared dye content on the impulse coupling coefficient is shown in Figure 7b. Under a laser energy density of 4.60 J·cm^−2^, the impulse coupling coefficient decreases with an increasing infrared dye content. For the liquid propellant with 0.3 wt.% dye content, the impulse coupling coefficient is 208.23 μN·s·J^−1^, which decreases to 170.14 μN·s·J^−1^ when the content increases to 0.4 wt.%. At a laser energy density of 11.03 J·cm^−2^, the impulse coupling coefficient is lowest at 262.36 μN·s·J^−1^ for the liquid propellant with a 0.4 wt.% dye content. As the content increases, the impulse coupling coefficient rises, reaching a maximum of 311.61 μN·s·J^−1^ at 0.6 wt.%. At higher laser energy densities (16.59 J·cm^−2^ and 21.60 J·cm^−2^), the impulse coupling coefficient of the liquid propellant increases with a higher infrared dye content. Under a laser energy density of 21.60 J·cm^−2^, the impulse coupling coefficients for liquid propellants with different dye contents are 333.55 μN·s·J^−1^, 524.44 μN·s·J^−1^, 542.85 μN·s·J^−1^, and 681.63 μN·s·J^−1^, respectively.

The relationship between the specific impulse and ablation efficiency of the ADN-based liquid propellant with varying infrared dye contents is shown in Figure 7c,d, respectively. At low laser energy densities, the infrared dye content has a minimal effect on the specific impulse and ablation efficiency of the liquid propellant. However, at higher laser energy densities, both the specific impulse and ablation efficiency increase with a higher infrared dye content. At a laser energy density of 21.60 J·cm^−2^ and an infrared dye content of 0.6 wt.%, the specific impulses are 26.43 s, 41.86 s, 43.56 s, and 54.43 s, with corresponding ablation efficiencies of 4.32%, 10.82%, 11.69%, and 18.21%.

The study results indicate that, at low laser energy densities, the infrared dye content has a minimal impact on the propulsion performance parameters of the liquid propellant, as the degree of ablation is relatively low. At higher laser energy densities, the degree of ablation increases, and the propulsion performance parameters significantly improve with a higher infrared dye content. Under the same laser energy density, a higher infrared dye content results in a smaller absorption depth for the liquid propellant, meaning a shorter laser propagation distance. Consequently, a higher infrared dye content allows the liquid propellant to absorb more laser energy per unit volume, leading to a greater degree of ablation. Therefore, at higher laser energy densities, the propulsion performance of the liquid propellant is significantly enhanced with an increased infrared dye content. Additionally, as an energetic liquid propellant, the ADN-based propellant undergoes dissociation and releases a certain amount of chemical energy when ablated by laser at high energy densities. The higher the infrared dye content and laser energy density are, the more chemical energy is released.

We summarised the results of the related literature studies into Table 2. From the specific impulse results, the propellant in this paper can obtain higher specific impulse values at the same energy density. All three studies obtain the highest impulse coupling coefficient at lower laser energy density, while the results of this study show that the impulse coupling coefficient increases with the increase in laser energy density. This is because the chemical reactions in this paper react fully with a higher laser energy. It also shows that the results of this paper are favourable for obtaining more stable propulsion performance by adjusting the laser energy density in a larger energy range. The literature [13,14] has added constraints in the experiments, so the impulse coupling coefficients obtained do not have much significance in direct comparison with this paper.

### 3.3. Ablation Flow Field Analysis

**Figure 8:**
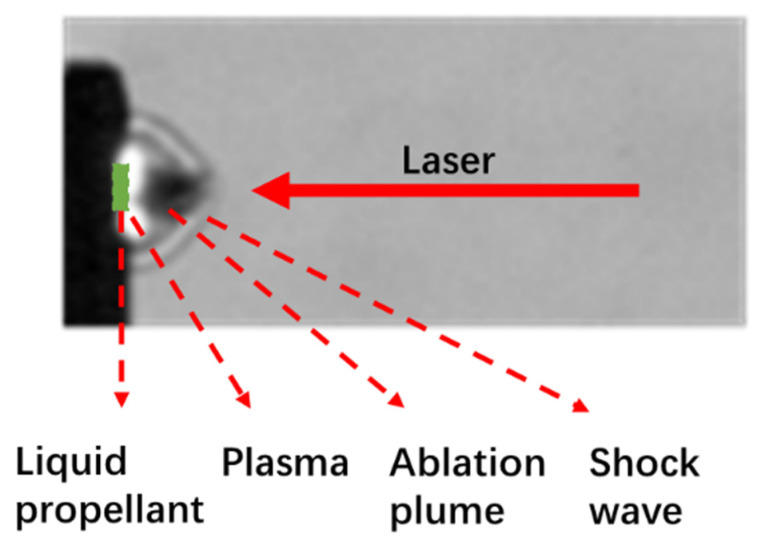
Typical laser ablation of a liquid propellant schlieren image (IR dye content 0.6 wt.%, 23.90 J·cm^−2^, 2.5 μs).

**Figure 9:**
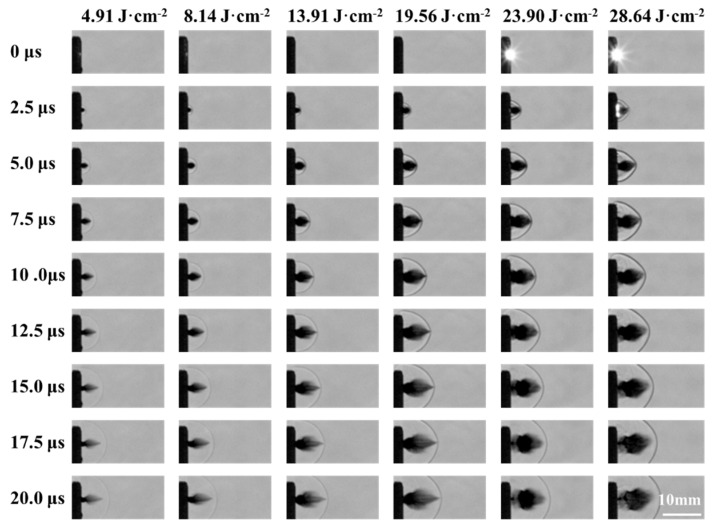
Shock wave evolution images of aqueous ADN solutions ablated by laser at different energy densities (IR dye content 0.6 wt.%).

The high-temperature, high-pressure plasma generated during the laser ablation of a liquid propellant expands supersonically to compress the surrounding air medium, forming a shock wave. The typical laser ablation of liquid propellant shock wave image is shown in Figure 8 and Figure 9, in which the infrared dye is 0.6 wt.%, the laser energy density is 23.90 J·cm^−2^. Laser irradiation to the surface of the liquid propellant and in the higher laser energy density can produce a plasma flash phenomenon. Figure 8 is a typical Schlieren image of the laser ablation of a liquid propellant. A clear shock wavefront is observed, and the shape of the wavefront is approximately semicircular. Bright areas are observed after the shock wave front, and the reason for the post-wave bright areas is that during the propagation of the shock wave along the direction of the incident laser, the density of the air medium after the wave decreases, and the transmittance to the light source of the pattern increases, resulting in the post-wave bright areas.

The shock wave evolution of the ADN solution with a 0.6 wt.% IR dye content under the action of laser ablation with different energy densities is shown in Figure 9, with a loading depth of 200 μm. At the moment of 0 μs, the laser irradiated on the surface of the liquid propellant and plasma flash phenomenon could be generated when the laser energy density was higher. At the moment of 2.5 μs, the flash phenomenon basically disappeared, and then a clear shock wave could be observed. As the time goes on, the ablation plume and the shock wave move along the direction of laser incidence, and the plume length and the radius of the shock wave increase continuously. When the laser energy density is higher, the ablation effect between the laser and the liquid mass is stronger, resulting in a stronger plasma and a larger radius of the shock wave. In addition, due to the plasma expansion and compression, the density of the medium at the surface of the shock wave array is higher, and the density of the air medium after the wave decreases, which leads to the lower light intensity at the surface of the shock wave array and th ehigher light intensity at the lower density after the wave.

**Figure 10:**
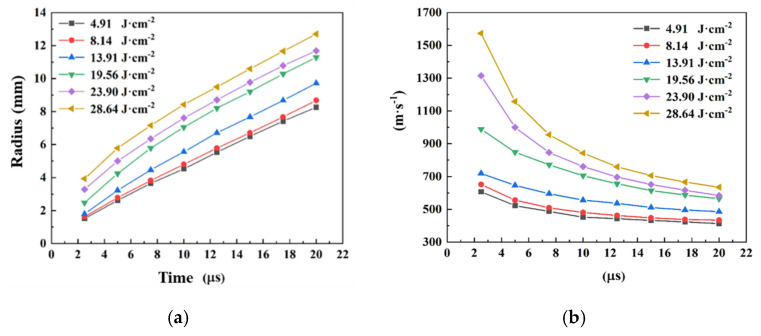
Shock wave radius (**a**) and shock wave velocity (**b**) of ADN aqueous solution ablated by laser with different energy densities versus time (IR dye content 0.6 wt.%).

The shock wave radius and velocity of ADN solutions subjected to laser ablation at different energy densities are shown in Figure 10. It is found that the shock wave radius of the ADN solution increases with time, while the average shock wave velocity decreases with time. With a laser energy density of 19.56 J·cm^−2^, the shock wave radius at 2.5 μs was about 2.47 mm, corresponding to an average shock wave velocity of 988.8 m·s^−1^. At 5 μs, the shock radius was 4.24 mm, and the average velocity was 848.3 m·s^−1^; at 20 μs, the radius of the shock wave increased to 11.29 mm, and the average shock wave velocity decreased to 564.6 m·s^−1^. The results show that the velocity of the shock wave decreases with time; because of the resistance of the background gas and the motion of the background gas caused by the shock wave propagate in the air, the energy of the shock wave is constantly lost and converted into the internal and kinetic energies of the background gas. The energy of the shock wave is constantly attenuated, which results in the decrease in the velocity of the shock wave with time.

The higher laser energy density will generate the larger radius of the shock wave, also with the higher velocity. When the laser energy density is 4.91 J·cm^−2^, the radius of the shock wave is small and the velocity is low, with the radius of the shock wave being 1.52 mm and the velocity being 606.7 m·s^−1^. When the laser energy density is 8.14 J·cm^−2^, the radius of the shock wave and the velocity are 1.63 mm and 651.7 m·s^−1^, respectively. When the laser energy density is increased to 28.64 J·cm^−2^, the radius of the shock wave of the liquid propellant and the velocity of the liquid propellant increase to 3.93 mm and 1573.0 m·s^−1^, respectively. The results show that the irradiated liquid matrix absorbs more laser energy when the laser energy density is higher, resulting in a higher ablation degree of the liquid matrix. The higher temperature and pressure of the plasma produced by the high energy density laser provides higher kinetic energy for the formation and motion of the shock wave, which results in a larger shock wave radius and higher shock wave velocity.

Overall, significantly luminous plasma appears in the ablation plume at higher laser energy densities, accompanied by higher velocity plasma shock waves. Infrared dyes play an important role in modulating the absorption depth of laser. However, the dye cannot be added too much or it will affect the energy content of the liquid propellant. Of course, the premise is that the chemical energy of the propellant can be fully released, which is affected by the laser energy density, the coupling efficiency of the laser pulse with the liquid propellant in space, and other factors. Compared to the factor of the infrared dye content, the laser energy density contributes more to the ablation efficiency, especially when the increase in laser energy density promotes the full release of chemical energy from the liquid propellant [23], which, in turn, also enhances the impulse, impulse coupling coefficient, and the plasma detonation velocity.

## 4. Conclusions

In this paper, the exothermic, light-absorbing, and propulsive properties of the ADN-based liquid propellant were researched using the liquid propellant laser-absorbing performance test system and the micro-impulse test system, and the effects of the laser energy density and the infrared dye content on the laser ablation propulsion performance of ADN-based liquid working media were investigated, and the conclusions of the study are as follows:

(1) The addition of infrared dye to the ADN-based liquid matrix can significantly improve the light absorption performance of the liquid matrix, and the absorption coefficient of the liquid matrix is 463.85 cm^−1^ with an absorption depth of 21.56 μm at an infrared dye content of 0.6 wt.%;
(2) The propulsive properties of the ADN-based liquid propellant increased with the laser energy density. At a laser energy density of 21.60 J·cm^−2^, the impulse is 74.22 μN·s, the impulse coupling coefficient is 681.63 μN·s·J^−1^, the specific impulse is 54.43 s, and the ablation efficiency is 18.21%.
(3) Significantly luminous plasma appears in the ablation plume at higher laser energy densities, accompanied by higher-velocity plasma shock waves. Compared to the factor of the infrared dye content, the laser energy density contributes more to the ablation efficiency, especially when the increase in laser energy density promotes the full release of chemical energy from the liquid propellant, which, in turn, also enhances the impulse, impulse coupling coefficient, and the plasma detonation velocity.

The result could be used for laser plasma micro-propulsion for micro-spacecraft, especially where there is a high demand for the energy density of liquid propellants.

## Figures and Tables

**Table 1:** Absorption coefficients of different infrared dyes at 1064 nm and solubility properties in water.

Parameters	IR1036	IR1064	IR1075	IR1089	IR-H
Absorption coefficients (cm^−1^)	92.18	88.33	96.52	74.37	56.04
Solubility properties in water	Soluble	Slightly soluble	Slightly soluble	Slightly soluble	Slightly soluble

**Table 2:** A comparative table of results of laser ablation liquid propellants including related results under similar laser pulses.

Propellants	Laser Densities/J·cm^−2^	Laser Absorption Depth/μm	Impulse /μN·s	Impulse Coupling Coefficient/μN·s·J^−1^	Specific Impulse/s	References
ADN, [AMIM][DCA], IR1036, water	4.6–21.6	40.20–21.56	3.73–74.22	170.14–681.63	2.73–43.86	Our work
Glyceron, 1% Carbon power	2.4–11.6	-	2–38	1250–67	-	[13]
Glycerol, 3% Carbon power (with a confinement)	8–58	-	-	3400–2200	5–18	[14]
Water (confined by glass layer)	2.06–8.93	-	-	1720–381	4.2–8.9	[15]

## Data Availability

The original contributions presented in this study are included in the article. Further inquiries can be directed to the corresponding author.
